# Health anxiety among medical and non-medical university students in Palestine: associations with intolerance of uncertainty, daily phone use, and field of study

**DOI:** 10.1186/s40359-026-04751-8

**Published:** 2026-05-23

**Authors:** Ahmad Fasfoos, Muhammad Suliman Sunkrot, Islam Jadallah, Ahmad H. Id’eas, Mai Hoshieh, Tasneem Haytham, Saja Aljunaidi, Janaa Issa, Amro K. Al-Manasrah, Ahmad Ahmoud

**Affiliations:** Faculty of Medicine, Hebron University, Hebron, State of Palestine

**Keywords:** Health anxiety, Medical student syndrome, Short health anxiety inventory, Intolerance of uncertainty, University students, Palestine

## Abstract

**Background:**

Medical student syndrome (MSS) is commonly used to describe illness-related anxiety in academic settings, particularly anxiety linked to exposure to disease-related information. However, it remains unclear whether elevated health anxiety is actually more common among medical students than among students in other fields. This study examined SHAI-defined elevated health anxiety among university students in Palestine and assessed its associations with intolerance of uncertainty, daily phone use, and field of study.

**Methods:**

A cross-sectional study was conducted among 484 university students in Palestine using an online self-administered questionnaire. Elevated health anxiety was assessed using the main section of the Short Health Anxiety Inventory (SHAI; items 1–14), with a score of ≥ 18 used as a screening threshold. MSS was treated as a conceptual framework rather than as a distinct clinical diagnosis. Intolerance of uncertainty was measured using the 12-item Intolerance of Uncertainty Scale (IUS-12). Bivariate associations were assessed using chi-square and Mann–Whitney U tests. Independent associations were examined using Poisson regression with robust variance, and results were reported as adjusted prevalence ratios (PRs) with 95% confidence intervals (CIs).

**Results:**

Overall, 27.3% of students met the SHAI-based screening threshold for elevated health anxiety. The prevalence was lowest among human medicine students (19.7%) and was higher among non-health-related students (33.3%) and other health-related students (34.3%), indicating that SHAI-defined elevated health anxiety was more common among students outside human medicine. This pattern remained evident after adjustment: compared with human medicine students, prevalence was significantly higher among non-health-related students (adjusted PR = 1.597, 95% CI: 1.112–2.295, *p* = 0.011) and other health-related students (adjusted PR = 1.635, 95% CI: 1.129–2.369, *p* = 0.009). Intolerance of uncertainty remained the strongest independent correlate; each 5-point increase in IUS-12 score was associated with a 29% higher prevalence of elevated health anxiety (adjusted PR = 1.29, 95% CI: 1.19–1.40, *p* < 0.001), and greater daily phone use was also independently associated with higher prevalence (adjusted PR = 1.041, 95% CI: 1.003–1.080, *p* = 0.033). Camp residence was likewise associated with higher prevalence (adjusted PR = 1.838, 95% CI: 1.035–3.266, *p* = 0.038).

**Conclusions:**

SHAI-defined elevated health anxiety was common among Palestinian university students, but it was not highest among human medicine students. The prevalence was higher among students in non-health-related and other health-related disciplines. Because the SHAI measures general health anxiety rather than anxiety specifically triggered by exposure to medical knowledge, these findings should be interpreted as evidence that elevated health anxiety in university settings may extend beyond medical students rather than as a definitive test of Medical Student Syndrome as a distinct phenomenon.

## Introduction

Health anxiety is increasingly understood as a dimensional construct that ranges from transient concern about illness to persistent preoccupation with serious disease rather than a simple present-or-absent state [1, 2]. Individuals with elevated health anxiety often show heightened attention to bodily sensations, maladaptive interpretation of benign symptoms, and repeated reassurance-seeking [1]. The Short Health Anxiety Inventory (SHAI) was developed to assess these cognitive and emotional features of health anxiety, and subsequent evidence has shown that it has acceptable reliability and strong construct validity across clinical, medical, and non-clinical settings [1, 2].

Within academic environments, this pattern of illness-focused worry is often discussed using the term medical student syndrome (MSS), which refers to the tendency of students to fear or identify with illnesses they study or repeatedly encounter in health-related material [3]. Although MSS is not a formal psychiatric diagnosis, the term remains useful as a descriptive label for a recognizable pattern of illness-related anxiety in educational settings [3]. Importantly, available evidence does not support the assumption that such concerns are exclusive to medical students, suggesting instead that MSS-related symptoms may reflect broader health-anxiety processes that can extend beyond medicine alone [3].

A particularly relevant mechanism in this context is intolerance of uncertainty (IU). IU refers to a dispositional tendency to perceive uncertainty as distressing, aversive, or unacceptable, and it has been increasingly recognized as a transdiagnostic mechanism across emotional disorders [4, 5]. This construct is directly relevant to illness-focused anxiety because ambiguous bodily sensations and incomplete health-related knowledge create exactly the kind of uncertainty that vulnerable individuals may find difficult to tolerate [4, 5]. Consistent with this interpretation, intolerance of uncertainty has been shown to be associated with hypochondriacal concerns even when examined alongside worry and obsessive–compulsive symptoms [5].

Digital behavior may also be relevant to SHAI-defined elevated health anxiety. University students now spend substantial parts of daily life on smartphones, and problematic or excessive smartphone engagement has been linked to adverse mental-health correlates in student and youth populations [6, 7]. In Palestine, smartphone addiction among medical students has also been associated with depression, anxiety, and stress [8]. These findings make daily phone use a plausible behavioral correlate of elevated health anxiety, particularly in student populations that are continuously exposed to digitally mediated information and repeated symptom-related content [6–8].

The problem is that elevated health anxiety remains conceptually discussed far more often than it is directly studied, especially outside narrowly defined medical-student samples. In the Palestinian context, published work has already shown that anxiety symptoms are common among medical students [9]. However, the available local literature has focused mainly on general anxiety symptoms or broader technology-related distress rather than on elevated health anxiety itself [8, 9].

This leaves an important gap in knowledge. To our knowledge, no study has specifically examined SHAI-defined elevated health anxiety among Palestinian university students across different fields of study, while simultaneously evaluating the role of intolerance of uncertainty and daily phone use within the same analytic framework. This gap matters because the phenomenon may not be confined to human medicine students alone; it may instead represent a broader student mental-health pattern shaped by uncertainty-related cognition and contemporary behavioral habits. Clarifying this issue is important not only for theory, but also for identifying which student groups may be more vulnerable and which psychological or behavioral correlates deserve attention in prevention and support efforts.

Accordingly, the present study examined elevated health anxiety among university students in Palestine using the main section of the SHAI. The concept of Medical Student Syndrome was used as a contextual framework for discussing illness-related anxiety in academic settings, but the measured outcome was general health anxiety rather than exposure-specific MSS. The study further evaluated the role of intolerance of uncertainty and selected behavioral and academic factors, with particular attention to daily phone use and field of study. By doing so, the study aimed to determine whether elevated health anxiety is more strongly related to field of study itself or to broader cognitive and behavioral vulnerability.

## Methods

### Study design and setting

This study used a cross-sectional design to examine the prevalence of SHAI-defined elevated health anxiety and its associated factors among university students in Palestine. Data were collected over a three-month period, from December 2025 to February 2026.

### Sample size

The minimum required sample size was estimated using the standard single-population proportion formula, assuming a 95% confidence level, a 5% margin of error, and a conservative expected proportion of 50% because no prior local estimate was available for the prevalence of SHAI-defined elevated health anxiety among university students in Palestine. This yielded a minimum required sample of 384 participants. The final analytic sample consisted of 484 students, exceeding the estimated minimum sample size.

### Study population and recruitment

The target population comprised students enrolled in Palestinian universities during the study period. Data were collected using an online self-administered questionnaire. Participants were recruited using a non-probability convenience sampling approach. Students were eligible if they were currently enrolled in a university in Palestine and completed the items required to construct the study variables. A total of 484 students were included in the final analysis. Because recruitment was conducted online and relied on voluntary participation, the sample should not be interpreted as statistically representative of all Palestinian university students.

### Study variables

#### Sociodemographic, academic, and behavioral variables

The questionnaire collected data on age, sex, place of residence, marital status, academic year, grade point average (GPA), field of study, self-reported average daily phone use in hours, regular exercise, smoking status, and health service sector. Daily phone use was assessed as the average number of hours spent using a phone per day and did not distinguish between different types of phone activity. For the main analysis, academic year was grouped into years 1–3 and years 4–6, and GPA was categorized as < 80 and ≥ 80. Field of study was grouped into non-health-related students, human medicine students, and other health-related students.

### SHAI-defined elevated health anxiety

Elevated health anxiety was assessed using the Short Health Anxiety Inventory (SHAI), a widely used self-report measure developed to assess the cognitive and emotional dimensions of health anxiety [1, 2]. The SHAI has demonstrated acceptable reliability and construct validity across clinical and non-clinical populations [1, 2]. In the present study, only the main section of the SHAI (items 1–14) was used. Scores on this section range from 0 to 42, with higher scores indicating higher levels of health anxiety. For the primary analysis, elevated health anxiety was defined as a SHAI main-section score ≥ 18, consistent with previous student-based research that used this threshold to identify health-related anxiety among medical students [10]. However, this cutoff has not been specifically validated among Palestinian or Arabic-speaking university students; therefore, it should be interpreted as a screening threshold rather than a clinical diagnostic cutoff. The SHAI measures general health anxiety and does not directly assess anxiety specifically triggered by exposure to medical knowledge. Accordingly, Medical Student Syndrome was used in this study as a conceptual framework rather than as a directly measured syndrome.

### Intolerance of uncertainty

Intolerance of uncertainty was measured using the 12-item Intolerance of Uncertainty Scale (IUS-12). The IUS-12 is a shortened form of the original scale and was developed to assess reactions to uncertainty, ambiguity, and future-oriented unpredictability [11]. It has demonstrated strong psychometric properties and is commonly analyzed as a total score, with higher values indicating greater intolerance of uncertainty [11, 12]. In the present study, the IUS-12 total score was treated as a continuous predictor.

### Ethical considerations

The study was conducted in accordance with accepted ethical standards for human research. Ethical approval was obtained from the Institutional Review Board of the Faculty of Medicine, Hebron University. Participation was voluntary, and informed consent was obtained electronically before questionnaire completion. No directly identifying personal information was collected.

### Statistical analysis

Data were analyzed using IBM SPSS Statistics version 26. Categorical variables were summarized using frequencies and percentages, while continuous variables were summarized using medians and interquartile ranges (Q1–Q3) because the main outcome-related variables were analyzed using non-parametric methods at the bivariate level.

The prevalence of SHAI-defined elevated health anxiety was calculated for the total sample and separately across the three field-of-study groups. Bivariate associations with elevated health anxiety were assessed using chi-square tests for categorical variables and Mann–Whitney U tests for continuous variables, as appropriate.

To identify factors independently associated with SHAI-defined elevated health anxiety, a Poisson regression model with robust variance was fitted, and results were reported as adjusted prevalence ratios (PRs) with 95% confidence intervals (CIs). This approach was selected because, in cross-sectional studies with binary outcomes, direct estimation of the prevalence ratio is generally preferable to the odds ratio, which may overestimate associations when the outcome is not rare [13, 14]. Variables that showed statistically significant associations with elevated health anxiety in the bivariate analysis were entered into the multivariable Poisson regression model. The final model included sex, GPA category, place of residence, field of study, age, daily phone use, IUS-12 total score, academic stage, and regular exercise. A two-sided p-value < 0.05 was considered statistically significant.

## Results

### Descriptive characteristics of the study sample

As shown in Table 1, a total of 484 university students were included in the analysis. The median age was 21 years (Q1–Q3: 20–22). Females constituted 65.3% of the sample, and most participants were single (94.6%). More than half of the students (56.8%) were in academic years 1–3, whereas 43.2% were in years 4–6. In terms of academic performance, 37.1% of students had a GPA below 80, while 62.9% had a GPA of 80 or higher. With respect to residence, 56.6% lived in cities, 38.6% in villages, and 4.8% in camps.

**Table 1:** Sociodemographic and academic characteristics of the participants

Characteristic	Category/Statistic	Value
Age (years)	Median (Q1-Q3)	21 (20–22)
Sex	Male	168 (34.7%)
	Female	316 (65.3%)
Marital status	Single	458 (94.6%)
	Married	26 (5.4%)
Academic stage	Years 1–3	275 (56.8%)
	Years 4–6	209 (43.2%)
GPA category	< 80	174 (37.1%)
	> = 80	295 (62.9%)
Place of residence	City	274 (56.6%)
	Village	187 (38.6%)
	Camp	23 (4.8%)

Continuous variables are presented as median (Q1-Q3). Categorical variables are presented as n (%). GPA category was available for 469 participants

Behavioral, service-related, and field-of-study characteristics are presented in Table 2. The median daily phone use was 5 h (Q1–Q3: 4–7). Most participants were non-smokers (90.9%). More than half of the sample (57.9%) reported using mixed governmental/private health services, while 20.2% used governmental services only and 21.9% used private services only. Regarding field of study, 46.1% were human medicine students, 31.6% were non-health-related students, and 22.3% were students from other health-related disciplines.

**Table 2:** Behavioral, service-related, and field-of-study characteristics

Characteristic	Category/Statistic	Value
Daily phone use (hours)	Median (Q1-Q3)	5 (4–7)
Smoking status	Non-smoker	440 (90.9%)
	Smoker	44 (9.1%)
Health service sector	Governmental	98 (20.2%)
	Private	106 (21.9%)
	Mixed (governmental/private)	280 (57.9%)
Field of study	Non-health-related students	153 (31.6%)
	Human medicine students	223 (46.1%)
	Other health-related students	108 (22.3%)

Continuous variables are presented as median (Q1-Q3). Categorical variables are presented as n (%)

### Prevalence of SHAI-defined elevated health anxiety

**Fig. 1:**
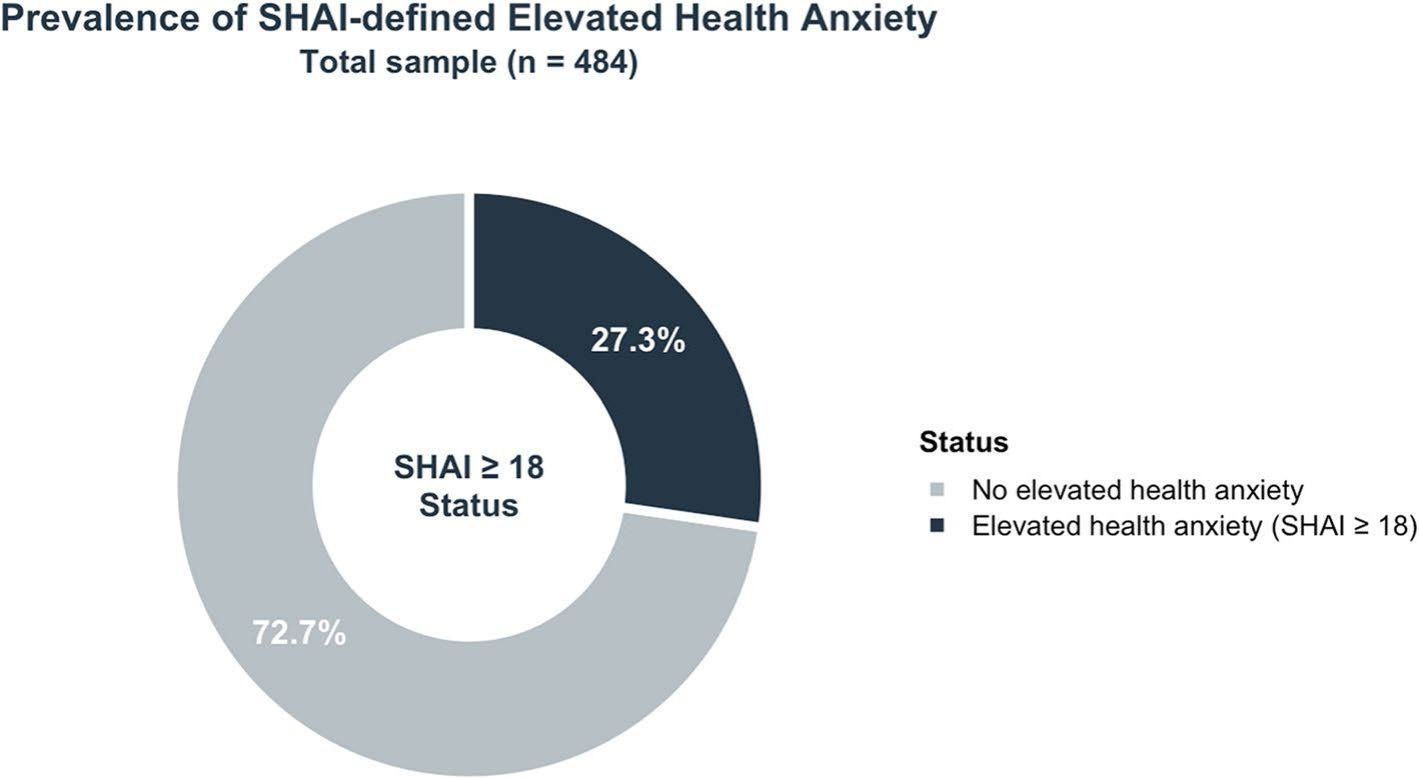
Prevalence of SHAI-defined elevated health anxiety in the study sample

As illustrated in Fig. 1, 132 of 484 students met the SHAI-based screening threshold for elevated health anxiety, corresponding to an overall prevalence of 27.3%.

When prevalence was examined by field of study, notable variation was observed. As shown in Table 3, the prevalence was lowest among human medicine students (19.7%), whereas it was higher among non-health-related students (33.3%) and other health-related students (34.3%). These findings indicate that elevated health anxiety was not concentrated among human medicine students and was, instead, more common in the other two academic groups.

**Table 3:** Bivariate associations with SHAI-defined elevated health anxiety

Characteristic	Category	No elevated health anxiety	Elevated health anxiety	*p*-value
Sex	Male	132 (78.6)	36 (21.4)	0.035
	Female	220 (69.6)	96 (30.4)	
Place of residence	City	194 (70.8)	80 (29.2)	0.017
	Village	146 (78.1)	41 (21.9)	
	Camp	12 (52.2)	11 (47.8)	
Marital status	Single	331 (72.3)	127 (27.7)	0.344
	Married	21 (80.8)	5 (19.2)	
Academic stage	Years 1–3	190 (69.1)	85 (30.9)	0.039
	Years 4–6	162 (77.5)	47 (22.5)	
GPA category	< 80	118 (67.8)	56 (32.2)	0.046
	≥ 80	225 (76.3)	70 (23.7)	
Hypertension	No	343 (73.4)	124 (26.6)	0.062
	Yes	9 (52.9)	8 (47.1)	
Diabetes mellitus	No	346 (72.7)	130 (27.3)	0.884
	Yes	6 (75.0)	2 (25.0)	
Regular exercise	No	282 (70.3)	119 (29.7)	0.009
	Yes	70 (84.3)	13 (15.7)	
Health service sector	Governmental	68 (69.4)	30 (30.6)	0.420
	Private	74 (69.8)	32 (30.2)	
	Mixed (governmental/private)	210 (75.0)	70 (25.0)	
Smoking status	Non-smoker	323 (73.4)	117 (26.6)	0.287
	Smoker	29 (65.9)	15 (34.1)	
Field of study	Non-health-related students	102 (66.7)	51 (33.3)	0.003
	Human medicine students	179 (80.3)	44 (19.7)	
	Other health-related students	71 (65.7)	37 (34.3)	
Age (years)	Median (Q1–Q3)	21 (20–23)	20 (19–22)	0.050
Daily phone use (hours)	Median (Q1–Q3)	5 (4–7)	6 (5–8)	< 0.001
IUS-12 total score	Median (Q1–Q3)	33 (27–38)	39 (33–46)	< 0.001

Categorical variables are presented as n (%), whereas continuous variables are presented as median (Q1–Q3)

### Bivariate analysis of SHAI-defined elevated health anxiety

The bivariate associations with SHAI-defined elevated health anxiety are presented in Table 3. Sex was significantly associated with the outcome, with elevated health anxiety being more prevalent among females than males (30.4% vs 21.4%, *p* = 0.035). Place of residence was also significantly related to elevated health anxiety (*p* = 0.017), with the highest prevalence observed among students living in camps (47.8%), followed by those living in cities (29.2%) and villages (21.9%).

A significant association was also found for academic stage. Students in years 1–3 showed a higher prevalence of elevated health anxiety than those in years 4–6 (30.9% vs 22.5%, *p* = 0.039). GPA category was similarly associated with the outcome, as students with GPA < 80 had a higher prevalence than those with GPA ≥ 80 (32.2% vs 23.7%, *p* = 0.046). Regular exercise showed an inverse relationship with elevated health anxiety, with lower prevalence among students who exercised regularly compared with those who did not (15.7% vs 29.7%, *p* = 0.009). Field of study was also significantly associated with the outcome (*p* = 0.003), with the lowest prevalence observed among human medicine students.

In contrast, marital status (*p* = 0.344), diabetes mellitus (*p* = 0.884), health service sector (*p* = 0.420), and smoking status (*p* = 0.287) were not significantly associated with elevated health anxiety. The association with hypertension approached significance but did not reach the conventional threshold (*p* = 0.062).

Age also differed marginally according to elevated health anxiety status. Students with elevated health anxiety had a lower median age than those without the outcome (20 [19–22] vs 21 [20–23], *p* = 0.050). Daily phone use was higher among students with elevated health anxiety than among those without it (6 [5–8] vs 5 [4–7] hours, *p* < 0.001). Similarly, IUS-12 total score was higher among students with elevated health anxiety (39 [33–46] vs 33 [27–38], *p* < 0.001), indicating a clear bivariate relationship between greater intolerance of uncertainty and the presence of the outcome.

### Multivariable analysis of SHAI-defined elevated health anxiety

The multivariable Poisson regression model with robust variance is presented in Table 4. After adjustment, IUS-12 total score remained strongly associated with elevated health anxiety. When expressed per 5-point increase for interpretability, IUS-12 score was associated with a 29% higher prevalence of elevated health anxiety (adjusted PR = 1.29, 95% CI: 1.19–1.40, *p* < 0.001).

**Table 4:** Multivariable Poisson regression with robust variance for factors associated with SHAI-defined elevated health anxiety

Predictor	Category	Adjusted PR	95% CI	*p*-value
Age (years)	Per 1-year increase	1.011	0.885 to 1.155	0.871
Sex (ref: Male)	Female	1.074	0.761 to 1.516	0.684
GPA category (ref: < 80)	≥ 80	0.910	0.685 to 1.209	0.516
Place of residence (ref: Village)	City	1.276	0.931 to 1.750	0.130
	Camp	1.838	1.035 to 3.266	0.038
Academic stage (ref: Years 1–3)	Years 4–6	0.926	0.579 to 1.483	0.750
Regular exercise (ref: No)	Yes	0.589	0.340 to 1.022	0.060
Daily phone use (hours)	Per 1-h increase	1.041	1.003 to 1.080	0.033
Field of study (ref: Human medicine students)	Non-health-related students	1.597	1.112 to 2.295	0.011
	Other health-related students	1.635	1.129 to 2.369	0.009
IUS-12 total score	Per 5-point increase	1.290	1.193 to 1.394	< 0.001

The overall model was significant (Likelihood ratio χ^2^ = 52.430, df = 10, *p* < 0.001). For interpretability, the IUS-12 association is reported per 5-point increase

*PR* Prevalence ratio, *CI* Confidence interval, *IUS-12* Intolerance of Uncertainty Scale–12

Daily phone use also remained independently associated with the outcome. Each additional hour of phone use per day was associated with a 4.1% increase in the prevalence of elevated health anxiety (adjusted PR = 1.041, 95% CI: 1.003–1.080, *p* = 0.033).

With regard to place of residence, living in a camp was independently associated with higher prevalence of elevated health anxiety compared with living in a village (adjusted PR = 1.838, 95% CI: 1.035–3.266, *p* = 0.038), whereas residence in a city was not significantly associated with the outcome (adjusted PR = 1.276, 95% CI: 0.931–1.750, *p* = 0.130). This estimate should be interpreted cautiously because the number of students residing in camps was small.

Field of study remained significant in the adjusted model. Compared with human medicine students, non-health-related students had a higher prevalence of elevated health anxiety (adjusted PR = 1.597, 95% CI: 1.112–2.295, *p* = 0.011), and other health-related students also had a higher prevalence (adjusted PR = 1.635, 95% CI: 1.129–2.369, *p* = 0.009).

Regular exercise showed an inverse, borderline association with elevated health anxiety (adjusted PR = 0.589, 95% CI: 0.340–1.022, *p* = 0.060). In contrast, sex, GPA category, age, and academic stage were not independently associated with the outcome after adjustment. The overall model was statistically significant (Likelihood ratio χ^2^ = 52.430, df = 10, *p* < 0.001), indicating that the included variables were jointly associated with elevated health anxiety within the sample.

## Discussion

### Main findings

This study showed that SHAI-defined elevated health anxiety was common among university students in Palestine, with 27.3% of participants meeting the predefined screening threshold. The prevalence was lowest among human medicine students and higher in both non-health-related students and other health-related students. In the adjusted model, intolerance of uncertainty remained the strongest correlate of elevated health anxiety, while daily phone use, camp residence, and field of study also remained independently associated with the outcome. By contrast, sex, age, GPA category, and academic stage were not independently associated after adjustment. These findings should be interpreted as evidence regarding general health anxiety measured by the SHAI rather than as a direct measurement of exposure-specific Medical Student Syndrome.

### Interpretation of findings

The strongest finding in this study was the robust association between intolerance of uncertainty and elevated health anxiety. This is theoretically coherent because intolerance of uncertainty has been described as a transdiagnostic mechanism across psychological difficulties [4], and because it has specifically been linked to hypochondriacal concerns and illness-focused worry [5]. Students who have more difficulty tolerating ambiguity may be more likely to interpret bodily sensations in a threatening way and to remain distressed in the absence of clear reassurance [4, 5].

The independent association with daily phone use is also plausible. Systematic review evidence shows that problematic smartphone use is common among university students and is associated with multiple adverse psychological correlates [6], while broader meta-analytic evidence links problematic smartphone use in young people to poorer mental-health outcomes, including anxiety-related symptoms [7]. Palestinian evidence has also shown that smartphone addiction is positively associated with depression, anxiety, and stress among medical students [8]. Although our study assessed daily phone use rather than smartphone addiction severity, the direction of the finding is consistent with that broader literature [6–8]. Nevertheless, daily phone use in the present study was measured only by duration. The study did not distinguish between health-related searching, symptom checking, academic use, social media use, or entertainment. Therefore, this association should be interpreted as reflecting general digital exposure rather than cyberchondria or health-related online searching specifically.

The most conceptually important finding was that human medicine students had lower elevated health anxiety than both non-health-related students and other health-related students in the adjusted model. This result does not support the assumption that elevated health anxiety, as measured by the SHAI, is necessarily highest among human medicine students. Waterman and Weinman argued that the evidence supporting MSS as a uniquely medical-student phenomenon is weak [3]. One plausible explanation for our finding is that human medicine students may have more structured biomedical knowledge, which could improve symptom appraisal and reduce catastrophic misinterpretation. Another possible explanation is habituation, whereby repeated exposure to disease-related material may reduce fear-based interpretations over time. However, these mechanisms were not directly measured in the present study, and the SHAI does not directly assess anxiety triggered by exposure to medical knowledge. Therefore, this interpretation should remain cautious [3, 5].

The positive association observed for camp residence should be interpreted cautiously, but it is scientifically plausible. A recent scoping review concluded that Palestinians in the Occupied Palestinian Territories and Palestinian refugees face substantial mental-health burdens [15], and a recent study in Palestinian refugee camps in the West Bank documented considerable trauma-related psychological vulnerability [16]. Accordingly, the higher prevalence observed among students residing in camps may reflect a broader context of accumulated psychosocial strain rather than academic factors alone [15, 16]. Because camp-specific stressors were not directly measured and the number of camp residents in our sample was relatively small, this interpretation should remain cautious. Therefore, the camp-residence finding should be considered exploratory and hypothesis-generating rather than confirmatory.

The inverse, borderline association with regular exercise is also directionally plausible. Prospective meta-analytic evidence suggests that higher physical activity is associated with lower odds of later anxiety symptoms and anxiety disorders [17]. Thus, the present estimate may reflect a potentially protective relationship, although it did not reach conventional statistical significance and should therefore be interpreted as suggestive rather than definitive.

### Comparison with previous literature

The present findings are consistent with prior literature placing intolerance of uncertainty near the center of anxiety-related vulnerability [4, 5]. Our results extend that literature by showing that IU was not merely associated with distress in general, but remained the strongest independent correlate of elevated health anxiety in this student sample.

The observed association between daily phone use and elevated health anxiety is also broadly aligned with previous work on digital behavior and mental health. Systematic review evidence in university students supports the relevance of problematic smartphone use and related online behaviors [6], broader meta-analytic evidence links problematic smartphone use to poorer mental-health outcomes in young people [7], and Palestinian evidence shows similar associations among medical students [8]. The present study does not duplicate those findings exactly because it focused on daily phone use rather than addiction severity, but it moves in the same direction [6–8].

The field-of-study finding contributes something particularly important to the MSS literature. Waterman and Weinman questioned whether MSS should really be regarded as a uniquely medical-student phenomenon [3]. Our results support that more critical position, because human medicine students were not the group with the highest burden. Instead, both non-health-related students and other health-related students had higher adjusted prevalence of elevated health anxiety. This suggests that elevated health anxiety may be better understood as a broader student mental-health pattern shaped by uncertainty-related cognition and behavioral context rather than by medical training alone [3, 5].

In the Palestinian context, previous work has already shown that anxiety symptoms are common among medical students [9]. The present study complements that literature by shifting the focus from general anxiety symptoms to a more specific illness-focused anxiety pattern, and by examining this pattern across multiple academic groups within the same analytic framework. In that sense, the study adds conceptual specificity to the local student mental-health literature [9].

### Strengths and limitations

This study has several strengths. It examined elevated health anxiety in a broad university sample, allowing direct comparison among human medicine students, other health-related students, and non-health-related students. It also incorporated a psychologically central variable—intolerance of uncertainty—together with a relevant behavioral variable—daily phone use. In addition, multivariable modeling helped distinguish crude associations from those that remained after adjustment.

Several limitations should also be acknowledged. First, the cross-sectional design precludes causal inference. Second, the study relied on self-reported questionnaire data, which may be affected by reporting bias. Third, the online non-probability convenience sampling strategy limits representativeness and may have introduced self-selection bias, as students with greater interest in illness-related concerns may have been more likely to participate. Consequently, the prevalence estimate should be generalized cautiously. Fourth, the SHAI measures general health anxiety and does not directly assess anxiety specifically triggered by exposure to medical knowledge; therefore, Medical Student Syndrome was used as a conceptual framework rather than as a directly measured syndrome. Fifth, the SHAI cutoff of ≥ 18 has not been specifically validated among Palestinian or Arabic-speaking university students and should be interpreted as a screening threshold rather than a clinical diagnostic cutoff. Sixth, the study did not assess previous psychiatric history, pre-existing anxiety disorders, generalized anxiety disorder, or current psychological treatment. Seventh, daily phone use was measured only by duration and did not capture the type of phone activity, including symptom searching or health-related browsing. In addition, daily phone use was modeled as a continuous variable, and potential non-linear or threshold effects were not formally examined. Finally, the association with camp residence should be interpreted cautiously because the number of camp residents was small.

### Implications

These findings suggest that elevated health anxiety in university settings should not be framed too narrowly as a problem of human medicine students alone. Instead, the pattern appears more closely related to uncertainty-related cognitive vulnerability and behavioral context, particularly daily digital engagement. Practically, this means that student mental-health initiatives should not focus exclusively on medicine, but should also consider other health-related and non-health-related disciplines.

The strong association with intolerance of uncertainty suggests that interventions addressing how students respond to ambiguity, bodily sensations, and incomplete reassurance may be especially relevant. The association with daily phone use also indicates that technology-related habits deserve attention when universities address student well-being. At a minimum, the present findings support treating elevated health anxiety as a meaningful student mental-health issue rather than as a trivial anecdote or a stereotype attached only to medical students.

## Conclusion

In this sample of Palestinian university students, more than one-quarter met the SHAI-based screening threshold for elevated health anxiety. Human medicine students did not show the highest prevalence; instead, elevated health anxiety was more common among non-health-related students and students from other health-related disciplines. Intolerance of uncertainty remained the strongest independent correlate, and daily phone use was also associated with higher prevalence. Because the SHAI measures general health anxiety rather than anxiety specifically triggered by exposure to medical knowledge, these findings should be interpreted as evidence that elevated health anxiety may extend beyond medical students rather than as a definitive test of Medical Student Syndrome as a distinct phenomenon.

## Data Availability

The datasets used and/or analyzed during the current study are available from the corresponding author on reasonable request.
